# Oxygen Sensing and Viral Replication: Implications for Tropism and Pathogenesis

**DOI:** 10.3390/v12111213

**Published:** 2020-10-25

**Authors:** Peter Jianrui Liu, Peter Balfe, Jane A McKeating, Mirjam Schilling

**Affiliations:** Nuffield Department of Clinical Medicine, University of Oxford, Oxford OX3 7FZ, UK; peter.liu@ndm.ox.ac.uk (P.J.L.); peter.balfe@ndm.ox.ac.uk (P.B.); jane.mckeating@ndm.ox.ac.uk (J.A.M.)

**Keywords:** hypoxia, hyperoxia, HIF, PHD, 2OG, virus, tissue tropism

## Abstract

The ability to detect and respond to varying oxygen tension is an essential prerequisite to life. Several mechanisms regulate the cellular response to oxygen including the prolyl hydroxylase domain (PHD)/factor inhibiting HIF (FIH)-hypoxia inducible factor (HIF) pathway, cysteamine (2-aminoethanethiol) dioxygenase (ADO) system, and the lysine-specific demethylases (KDM) 5A and KDM6A. Using a systems-based approach we discuss the literature on oxygen sensing pathways in the context of virus replication in different tissues that experience variable oxygen tension. Current information supports a model where the PHD-HIF pathway enhances the replication of viruses infecting tissues under low oxygen, however, the reverse is true for viruses with a selective tropism for higher oxygen environments. Differences in oxygen tension and associated HIF signaling may play an important role in viral tropism and pathogenesis. Thus, pharmaceutical agents that modulate HIF activity could provide novel treatment options for viral infections and associated pathological conditions.

## 1. Introduction

**Figure 1:**
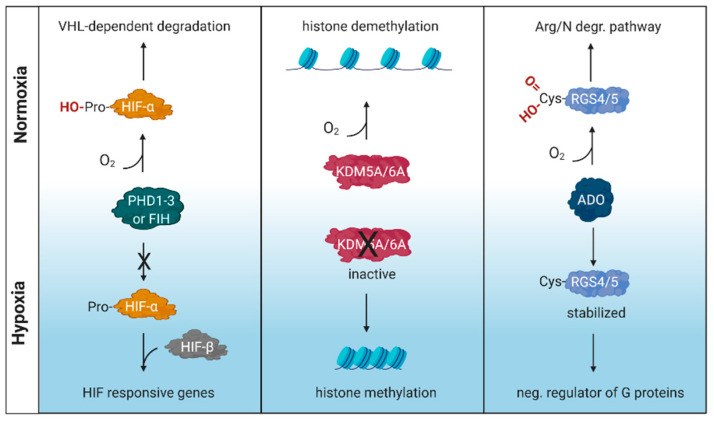
**Schematic illustration of oxygen sensing mechanisms.** These include the prolyl hydroxylase domain (PHD)/factor inhibiting HIF (FIH)-hypoxia inducible factor (HIF) pathway, lysine-specific demethylase (KDM) 5A and KDM6A pathways and the cysteamine (2-aminoethanethiol) dioxygenase (ADO) pathway. RGS, regulator of G protein signaling; VHL, von Hippel–Lindau tumor suppressor gene. Created with BioRender.com.

Oxygen is essential for survival and organisms have developed mechanisms to detect and respond to variable oxygen tensions. These include the prolyl hydroxylase domain (PHD)/factor inhibiting HIF (FIH)-hypoxia inducible factor (HIF) pathway [1,2] along with the more recently described cysteamine (2-aminoethanethiol) dioxygenase (ADO) pathway [3] and lysine-specific demethylase (KDM) 5A and KDM6A pathways [4,5] (Figure 1). PHDs and KDMs are members of a family of enzymes that are dependent on oxygen, Fe(II), ascorbate and the Krebs cycle intermediate 2-oxoglutarate (2OG), that regulate fundamental cellular processes by catalysing the hydroxylation or demethylation of DNA, RNA or proteins such as histones (reviewed in [6]).

The importance of HIFs in oxygen sensing was recognised by the 2019 Nobel Prize in Physiology or Medicine awarded to William Kaelin, Peter Ratcliffe and Gregg L. Semenza [7]. HIFs are heterodimeric transcription factors comprising an alpha and beta subunit that bind a consensus RCGTG(C) motif or hypoxic responsive element (HRE) in the promoter and enhancer regions of target genes. When oxygen is abundant, newly synthesised HIFα subunits, including HIF-1α and HIF-2α isomers, are hydroxylated by PHD or FIH, poly-ubiquitinated via von Hippel-Lindau factor (VHL) and targeted for proteasomal degradation. However, when oxygen is limiting these subunits are stabilised, translocate to the nucleus and dimerise with HIF-β to regulate a myriad of host target genes [1]. The PHD-HIF pathway is known to regulate genes with wide ranging functions, from metabolism and immunity, to DNA repair and carcinogenesis [8].

**Figure 2:**
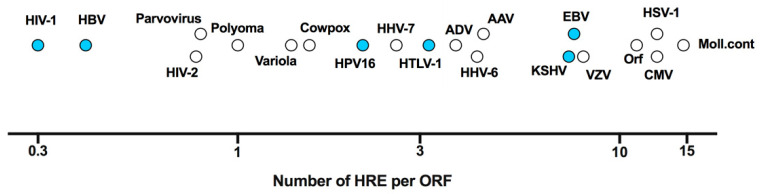
**Frequency of HIF response elements (HRE) in viral DNA genomes.** The frequency of HRE elements (RCGTG) is plotted against the number of open reading frames (ORF) for a range of DNA viruses with those marked in blue discussed in this review. Referent sequences presented were selected from Genbank as follows (accession numbers in brackets); adeno-associated virus (AAV, NC_001401.2), adenovirus (ADV, AC_000007.1), cytomegalovirus (CMV, KU317610.1), cowpox virus (CowPox, NC_003663.2), Epstein–Barr virus (EBV, NC_009334.1), hepatitis B virus (HBV, NC_003977.2), human herpesvirus 6 (HHV-6, NC_000898.1), HHV-7 (NC_001716.2), Kaposi’s sarcoma-associated herpesvirus (KSHV, NC_009333.1), human immunodeficiency virus type 1 (HIV-1, NC_001802.1), HIV-2 (NC_001722.1), human papillomavirus 16 (HPV16, NC_001526.4), herpes simplex virus type 1 (HSV-1, NC_001806.2), human T-lymphotropic virus 1 (HTLV-1, NC_001436.1), Molluscum Contagiosum (Moll.cont, NC_001731.1), Orf virus (Orf, NC_005336.1), parvovirus (NC_000883.2), polyomavirus (NC_031757.1), varicella-zoster virus (VZV, NC_001348.1) and variola virus (NC_001611.1).

As obligatory intracellular parasites, viruses depend on the host cellular machinery to replicate. Important factors that shape the cellular microenvironment include temperature, pH and oxygen tension. As variable oxygen tension regulates host gene expression, protein modification, metabolism and epigenetic regulation, it is not surprising that oxygen availability can influence multiple steps in the viral life cycle [4,9]. A wide range of oxygen tensions are found within different tissues and organs, ranging from <1% in the skin to 14.5% in arterial blood vessels (reviewed in [10]). However, the majority of in vitro studies on viral replication have been conducted at comparatively high atmospheric oxygen tension (18%) [11], where HIFs are not active and consequently their role in viral replication may have been overlooked. Viral infection, inflammatory responses and tissue damage induced reactive oxygen species (ROS) can all stabilise HIF expression, highlighting the complex interplay between oxygen sensing pathways and viral replication [12,13]. Viruses have either DNA or RNA genomes [14] and this can define whether HIFs regulate viral transcription by direct binding to the viral genome or via indirect regulation of host genes. Screening DNA genomes representing the major viral families shows an approximate 50-fold variation in the frequency of HREs relative to the number of open reading frames (ORFs), ranging from 0.3 for HIV-1 (3 HRE/10 ORFs) to 14.7 for Molluscum contagiosum (2395 HRE/163 ORFs) (Figure 2). These data are consistent with variable sensitivity of viruses to low oxygen environments.

**Figure 3:**
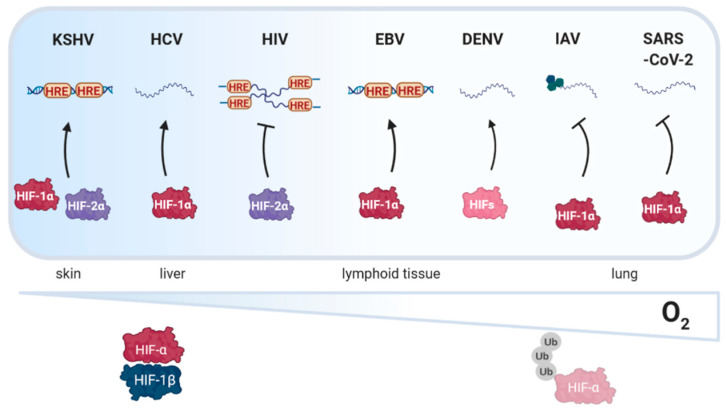
**Schematic illustration depicting how oxygen tension in different tissues affects viral replication.** Hypoxia inducible factor (HIF) signaling enhances (black arrow) the replication of Kaposi’s sarcoma associated herpesvirus (KSHV), hepatitis C virus (HCV), Epstein Barr virus (EBV) and dengue virus (DENV). In contrast, human immunodeficiency virus type I (HIV-1), influenza A virus (IAV) and severe acute respiratory syndrome coronavirus 2 (SARS-CoV-2) replication is dampened by HIF signaling. HRE, HIF response element, Ub, ubiquitin. Created with BioRender.com.

These data prompted us to review the current knowledge on how oxygen tension impacts viral replication (Figure 3) and how viruses manipulate the PHD-HIF pathway. Using a systems-based approach we discuss the literature in the context of viruses infecting and replicating in different tissue sites. Previous reviews have generally focused on the role of HIFs in viral carcinogenesis and host immunity [15,16,17,18]. Finally, we review pharmaceutical agents that modulate HIF activity and discuss their potential use as novel treatments for viral infections and associated pathological conditions [19,20,21].

## 2. The Skin and Epidermis

The skin plays an essential role in responding to environmental stimuli, where specific deletion of HIF-1α from the epidermis in mice inhibits renal erythropoietin (EPO) synthesis in response to hypoxia [22]. Furthermore, mice with an epidermal deletion of the VHL factor, a negative regulator of HIF, show increased EPO expression and polycythemia.

### 2.1. Kaposi’s Sarcoma Associated Herpesvirus (KSHV)

KSHV is a member of the *Herpesviridae* double-stranded DNA viruses that infects epithelial cells and keratinocytes, where the local oxygen tension is in the range of 1–2.5% [10,23,24]. KSHV causes Kaposi’s sarcoma, the most common acquired immunodeficiency syndrome related neoplasm [25] and viral lytic gene activation is associated with oncogenesis [26]. In 2003, Haque et al. identified functional HREs in the promoter regions of the ORF34 and Rta genes, which are involved in lytic switch, late stage gene expression and viral particle production [27,28]. Hypoxia activated both promoters and while ORF34 promoter was regulated by both HIF-1α and HIF-2α, the Rta promoter was preferentially regulated by HIF-2α. In 2006, Haque et al. went on to identify an additional HRE in the ORF35–37 promoter region that was co-regulated by both HIF-1α and HIF-2α [29]. These active HREs in the KSHV lytic gene promoters support an essential role for HIFs in regulating viral latency. KSHV-associated tumours are highly vascularised, a process which is partly mediated by virus-induced HIF signaling. Firstly, KSHV encoded G protein-coupled receptor (vGPCR) induces vascular endothelial growth factor (VEGF) expression and associated angiogenesis [25]. The vGPCR induces HIF-1α regulatory domain phosphorylation via p38/MAPK and mTOR pathways [30]. Secondly, the KSHV-encoded viral interferon (IFN) regulatory factor 3 (vIFR3) binds to HIF-1α and prevents its degradation [31]. Finally, the KSHV encoded latency-associated nuclear antigen acts as a transcriptional coactivator interacting with HIF-1α protein and enhances HIF-1α mRNA transcription [32,33]. These studies are consistent with the reported 34% overlap between KSHV infection and hypoxia gene expression profiles [34], suggesting that KSHV exploits the HIF signaling pathway and multiple redundant pathways to ensure HIF expression. Furthermore, inhibiting HIF in immunodeficient athymic mice reduced VEGF expression and tumour growth in a syngeneic mouse model [30]. KSHV-associated angiogenesis was potentiated by the hypoxia mimetic, deferoxamine mesylate [35], demonstrating an essential role for HIFs in KSHV pathogenesis. KSHV can also cause non-skin related cancers, however, the role of HIFs in these non-skin related cancers have not been studied.

### 2.2. Human Papilloma Virus (HPV)

HPV are a family of small double-stranded DNA viruses that infect basal epithelia and specific strains have been associated with a risk of oncogenesis [36,37]. The link between HPV strains 16 and 18 and cervical cancer has led to their classification as high risk and integration of HPV16 associates with HIF-1α overexpression [38]. HPV16 and 18 encoded E6 and E7 proteins stabilise HIF-1α and induce VEGF and IL-8 expression that is associated with increased angiogenesis [39,40,41]. Three studies have suggested alternative non-exclusive mechanisms for HPV to stabilise HIFs: (1) E6 prevents HIF-1α association with VHL [42]; (2) E6 and E7 induce HIF-2α protein via liver kinase B1 (LKB1) modulation [43] and (3) E2 binding to mitochondrial membrane components of the respiratory chain induce reactive oxygen species (ROS) [44]. There are limited clinical studies addressing the translational impact of HIFs in HPV associated cancers. However, in a cohort of patients with oropharyngeal squamous cell carcinoma there was some evidence for HIF-1α expression in the tumour associating with clinical outcome [45]. Jo et al. reported that elevated VEGF levels in a cohort of patients diagnosed with HPV16-associated oropharynx squamous cell carcinoma was independent of HIF-1α expression [46]. Given the technical limitations in staining for HIF-1α in tissue [47] and the oxygen rich environment of the oropharynx, ex vivo patient sample processing methods may require optimisation before interpreting these studies. Further clinical studies are required to fully examine the relationship between HPV, HIFs and patient outcomes.

## 3. The Liver

The liver is a naturally low oxygen environment with the highest oxygen tension near its periportal region at 8%, gradually decreasing to 4% in the pericentral area. A recent single cell sequencing study of the mouse liver [48] reported this oxygen gradient to associate with liver zonation, a phenomenon where hepatocytes show distinct functional and structural heterogeneity across the liver [49]. These infections are the leading cause for HCC worldwide and associated with significant mortality, accounting for more than 1.3 million deaths per year [50]. Hepatitis B and C viruses are a global health problem causing acute and chronic infections that can lead to liver cirrhosis and hepatocellular carcinoma (HCC).

### 3.1. Hepatitis C Virus (HCV)

HCV is a positive-sense single-stranded RNA virus in the *Flaviviridae* family that infects the liver. Low oxygen has been reported to increase HCV RNA levels in human hepatoma Huh-7 and HepG2 model systems [51,52]. These studies reported differences in the HIF-dependency of the low oxygen-increase in HCV RNA that most likely reflects the different oxygen tensions used in the experimental models: Vassilaki et al. [51] cultured the infected cells at 3% oxygen and showed a minimal role for HIFs in regulating HCV replication, whereas Wilson et al. [52] reported a positive role for HIFs in regulating HCV replication at 1% oxygen. Since the HIF-PHD and FIH degradation pathways are still functionally active at 3% oxygen there will be limited HIF transcriptional activity, highlighting the importance of the % oxygen applied in vitro model systems and how this impacts on HIF signalling. The study by Vassilaki highlights the role for hydroxylases within the 2OG oxygenase family in regulating HCV replication. A recent report showed the importance of N6-methyladenosine (m^6^A) modification in the replication of HCV and other *Flaviviridae* RNAs and viral particle genesis that was mediated by fat mass and obesity-associated protein (FTO), a member of the 2OG oxygenases [53].

The phospholipase autotaxin is hypoxic regulated and pharmacological inhibition or siRNA silencing of autotaxin-lysophosphatidic acid signaling reduced HCV replication, suggesting a role for HIFs to promote HCV replication via this pathway [54]. Autotaxin generates the biologically active lipid lysophosphatidic acid that plays a pro-tumorigenic role in a wide number of cancers. Farquhar et al. reported a positive association between hypoxic gene expression in HCV associated HCC tissue and autotaxin transcript levels [54]. Furthermore, the HCV encoded core [55,56,57,58] and E1E2 glycoproteins [52] stabilised HIF expression by inducing an unfolded protein-stress response. Further reports showing that HCV induction of NF-kB, STAT-3, PI3-K-aKT, and p42/44 MAPK signaling stabilised HIF-1α [54,59,60,61], highlight the multiple pathways for HCV induced HIF signaling. Nassimuzzaman et al. [61] reported HIF-dependent VEGF expression in HCV infected cells and suggested a role for HCV in activating angiogenesis. This observation was extended by Wilson et al. who showed that HCV-induced VEGF reduced hepatoma cell polarity and potentiated viral transmission [62] and increased epithelial-mesenchymal transition, suggesting a role for HCV stabilised HIFs in promoting fibrosis and liver injury.

### 3.2. Hepatitis B Virus (HBV)

HBV is a member of the *Hepadnaviridae* family of partially double-stranded DNA viruses that infect the liver. In contrast to our knowledge of HIFs in regulating HCV, currently there are no published studies exploring the role of HIFs in regulating HBV transcription. Hallez et al. reported that hypoxia induced human deoxyribonuclease 1 (DNASE1) could catabolise the encapsidated DNA genomes, resulting in a high frequency of empty or ‘light’ virions HBV [63]. The majority of experiments used cobalt chloride and dimethyloxalylglycine, mimetic agents that can inhibit prolyl hydroxylase enzymes and stabilise HIFs. However, these mimetics have been reported to induce additional oxygen-independent biological effects [64,65].

Several reports have studied the HBV encoded regulatory protein HBx that interacts with a myriad of host factors including HIF-1α and contributes to liver pathogenesis. Yoo et al. reported that HBx induced HIF-1α protein and mRNA upregulation through two independent mechanisms: (1) upregulation of MTA1 and HDAC1/2 that perturbed HIF-1α deacetylation and (2) prevention of HIF-1α association with VHL [66,67,68]. HBx was also reported to bind HIF-1α and increase its stability [69] and C-terminal truncation of HBx abrogated this association [70]. A significant limitation of these studies is their dependence on HBx overexpression systems. Lui et al. reported that a variety of in vitro and in vivo HBV model systems failed to show a role for HBx in stabilizing or modifying HIF transcriptional activity However, increased HIF target gene expression was observed in liver tissue from a chronic hepatitis B (CHB) cohort that associated with inflammatory immune responses [71]. Since inflammation and associated oxidative stress are known to induce HIF-transcription, the authors conclude that HBV-associated inflammation drives HIF expression.

Several clinical studies have reported an association between increased HIF expression and HBV-related disease outcome. Xie et al. showed that HIF-1α expression in HCC associated with shorter survival [72]. Similarly, Osman et al. demonstrated that HIF-1α expression in HCC associated with larger tumour size, multifocal malignancies and more advanced disease [73]. Genetic variation in HIF-1α has also been linked with HBV-HCC risk, where the CG haplotype associated with increased risk relative to the CA haplotype [74]. Furthermore, a HIF-2α single nucleotide polymorphism rs13419896 associated with an increased risk of liver cirrhosis [75]. Similarly, high KDM5B expression showed a negative association with HCC prognosis [76]. These clinical studies support translational studies to explore the application of HIF inhibitors to HBV related pathologies.

## 4. The Immune System

Lymphoid organs operate at oxygen levels in the range of 0.5–4.5% O_2_ [77,78,79], which are sufficient to activate the HIF pathway [80].

### 4.1. Human Immunodeficiency Virus Type I (HIV-1)

Human immunodeficiency virus type I (HIV-1) is a retrovirus with an RNA genome that is reverse transcribed into cDNA and integrated into the host genome where it serves as a transcriptional template. If the integrated provirus is methylated and transcriptionally silenced, the viral genome becomes latent. Reactivation of HIV from latent reservoirs is a crucial step of ‘shock-and-kill’ treatment approaches. HIV-1 primarily replicates in CD4^+^ T cells and the major virus reservoirs are thought to be in lymphoid tissues: hence this virus has evolved to replicate in cells that may experience widely differing oxygen tensions. Accumulating evidence shows that a hypoxic environment inhibits HIV replication and reactivation [81,82]. HIF-2α can repress HIV transcription under low oxygen conditions (1% O_2_) via a direct interaction with a conserved HRE in the U3 region of the long terminal repeat (LTR) [81]. In addition, hypoxic reduction in cyclin T1 activity was reported to reduce Tat mediated transcription [82]. Duette et al. reported the hypoxia mimetic cobalt chloride induced a modest 1.5 fold increase in HIV-1 replication [83], however, since these mimetics activate additional signaling pathways [64,65] further investigations are required to explore these differences.

Chronic immune activation, metabolic changes and inflammation during HIV-1 replication induce mitochondrial ROS [83]. The HIV-1 accessory protein Vpr was reported to increase HIF-1α expression via induction of ROS [84] and is consistent with an independent report showing that expression of Vpr in the monocytic cell line U937 increased HIF-dependent glycolysis [85]. In line with these findings, clinical studies reported HIF-1α expression in the brain of AIDS patients diagnosed with dementia [84] and increased HIF-2α and VEGF protein in kidney biopsies from patients with HIV-associated nephropathy [86]. Taken together, these data suggest that the hypoxic environment may be relevant to any successful shock-and-kill antiviral strategy.

### 4.2. Human T-Lymphotropic Virus Type 1 (HTLV-1)

The human T-lymphotropic virus type 1 (HTLV-1) is a retrovirus that integrates into the host genome. An estimated 5–10 million people worldwide are infected by HTLV-1 and in 5–10% of cases this leads to a CD4-T cell malignancy, known as adult T-cell leukemia or a progressive inflammatory disease of the spinal cord. In contrast to HIV-1, hypoxia enhanced HTLV-1 reactivation from latency via a HIF-independent process that involved the 2OG metabolite, suggesting a role for oxygenases in reactivating the genome [87]. Furthermore, there is evidence that the HLTV-1 encoded transactivator protein, Tax, induces HIF-1α protein expression and suppresses expression of the cellular proapoptotic BH3-only proteins Bim and Bid [88]. Since HTLV-1 may benefit from a hypoxic environment through several different mechanisms, a better understanding of these interactions could identify therapeutic targets.

### 4.3. Epstein Barr Virus (EBV)

Epstein Barr virus (EBV) is a double-stranded DNA virus in the family of *Herpesviridae* that replicates in CD20^+^ B cells and epithelial cells of the oropharynx and is associated with a number of malignancies, including Hodgkin’s lymphoma and Burkett’s Lymphoma [89]. As described for KSHV, HIF-1α directly binds the primary latent-lytic switch BZLF1 gene promoter, Zp, and activates transcription [90,91]. The EBV latent membrane protein 1 (LMP1), an oncogenic protein capable of B-cell immortalisation, increases HIF-1α protein expression by upregulating the Siah1 E3 ubiquitin ligase that degrades PHD1 and PHD3 [89,92,93,94]. In addition, the loss of VHL/HIF-1α complexes stabilises HIF-1α and associated HIF signaling, including pyruvate dehydrogenase kinase 1 (PDK1) and the pyruvate kinase M2 (PKM2) isoform, resulting in an increase in lactate production and glucose consumption [95].

There is limited evidence of an association between the survival of patients with EBV^+^ nasopharyngeal cancers and HIF-1α or VEGF expression [96]. Although LMP1 expression was noted in all EBV+ nasopharyngeal carcinomas, there was limited evidence of LMP1 co-localisation with HIF-1α. Despite the lack of a direct correlation between HIF expression and nasopharyngeal cancer clinical outcome, Yang et al. reported that LMP1 blockade increased the sensitivity of nasopharyngeal carcinoma to radiotherapy by downregulating HIF-1α and VEGF activity and decreasing phosphorylated JNKs/c-Jun signaling [97]. This observation was further validated through an in vivo experiment showing a significant reduction in tumour growth.

### 4.4. Dengue Virus (DENV)

DENV is a member of the *Flavivirus* genus that includes yellow fever, Zika, West Nile and Japanese encephalitis viruses. DENV is the most prevalent arbovirus disease in the world, with a spectrum of symptoms ranging from asymptomatic to severe haemorrhagic fever. DENV is transmitted by *Aedes aegypti* mosquito with potentially 3.9 billion people currently at risk of contracting infection [98]. Severe dengue fever most often occurs in secondary/tertiary infections and is thought to be mediated via antibody-dependent enhanced (ADE) infection along with a skewed T cell response. DENV primarily replicates in macrophages [99,100] and infection is associated with an oxidative stress response that plays a role in the immunopathology of the disease [101]. Gan et al. showed that hypoxia enhanced antibody-dependent DENV infection of THP-1 cells and primary human monocytes by two independent mechanisms [102]. Firstly, HIF-1α upregulated fragment crystallisable gamma receptor IIA (FcγRIIA). Secondly, membranous lipid concentrations were increased under hypoxia independently of HIF-1α. These processes increased antibody-opsonised DENV infection of monocytes. HIFs were also reported to promote DENV RNA replication and translation through a HIF-1α dependent mechanism in Huh-7 hepatoma cells [103]. DENV induces an oxidative stress response that may lead to HIF expression and hypoxic reprogramming [104]. Frakolaki et al. reported that DENV infection activated a HRE-luciferase reporter plasmid in Huh7 cells under atmospheric oxygen tension [103]. However, no further experiments were conducted to directly examine HIF protein levels or transcription of HIF target genes, limiting the conclusions of the study. Further studies are required to dissect the influence of physiological oxygen tension and tissue specific mechanisms on DENV regulation as well as the influence of DENV replication on local oxygen tension and metabolism.

## 5. The Respiratory Tract

Oxygen levels in the lung can vary in different locations within adults but also during development. Le et al. reported a median oxygen tension of 5.6% for adult lung tissue [105]. HIFs play an important role in fetal lung development and when the lung is hypoxic, loss of HIF expression can lead to impaired lung development or even death [106]. At the same time HIF upregulation due to lung damage, pulmonary hypertension or in lung cancer can negatively correlate with disease outcome.

### 5.1. Influenza Virus (IAV)

Influenza A virus (IAV) is a negative-sense single-stranded RNA virus associated with respiratory infections. IAV strains differ in their preference to infect the upper or lower respiratory tract [107]. Zhao et al. reported that IAV H1N1 (PR8) replicated to a higher titre in mice with targeted HIF-1α knockout in type II alveolar epithelial cells (AEC2) [108], accompanied by increased lung inflammation and mortality. Although the underlying mechanisms require further exploration, these data support an indirect role for HIF-1α in regulating IAV replication by inhibiting autophagy as a result of decelerated glycolysis. IAV has been reported to increase glycolysis by enhancing glucose uptake, lactate production and oxygen consumption rates [109,110]. Oral treatment with BEZ235 (a putative PI3 K/mTOR inhibitor) decreased glycolysis and reduced virus replication and mortality in a mouse model [111]. This observation contrasts with data from Zhao et al. [108] that may reflect cell-type dependent metabolic responses in the lung. It is likely that the role of HIF in influenza infection is more complicated as Zhang et al. recently showed that RIG-I like receptor (RLR) activation, a key sensor of IAV infection, suppresses glycolysis by inhibiting hexokinase [112]. HIF-1α is an important regulator of metabolic processes and is potentially regulated by immune responses against the virus. Indeed, HIF-1α induces proinflammatory cytokines and plays a key role in AEC2 differentiation and alveolar repair following IAV infection through activation of Notch signaling [113,114,115].

Supplemental oxygen (hyperoxia) given to premature infants associates with an increased risk of lung dysfunction and susceptibility to respiratory infections in adult life [116,117,118]. In a mouse model hyperoxia caused a significant reduction of pro-surfactant protein C-positive alveolar epithelial Type II cells by 8 weeks of age [116]. This finding is supported by a report identifying a protective role of superoxide dismutase to hyperoxia in a transgenic mouse model. Hence, oxygen tension and metabolic changes can impact the alveolar epithelial balance and response to injury. Further studies would elucidate the metabolic effects of HIF on different cell types in the lung, IAV replication, and whether oxygen tension affects IAV tissue tropism.

Infection with IAV leads to acute lung injury which activates hypoxia signaling and HIF induction. Several groups reported that IAV infection stabilises HIF-1α, although their proposed mechanisms vary. Zhao et al. showed an increase in HIF-1α expression in H1N1 (PR8) infected A549 cells with a modest increase in mRNA levels [108]. Ren et al. observed HIF-1α induction during H1N1 (PR8) infection as a result of reduced FIH expression [119]. Huo et al. reported no increase in HIF mRNA or protein levels, but an increase in nuclear translocation. Since IAV infection can result in a caspase-dependent enlargement of nuclear pores and non-specific protein uptake by the nucleus, this finding requires further validation [120]. Infection of the murine mastocytoma cell line P815 with different IAV strains (human H1N1, avian H5N1 and H7N2) showed that HIF-1α was activated by H7N2 but not by H1N1 or H5N1 [121], suggesting differing abilities of IAV strains to activate HIF-signalling pathways. It was interesting to note that H7N2 infection which activated HIF-signaling replicated to lower titre. Given the known effect of HIF-1α on degranulation and inflammatory factor production in immune cells, these differences could have wide-ranging implications [122]. HIF signaling is likely to be an important determinant for the outcome and recovery from IAV infections.

### 5.2. SARS-Coronavirus-2 (SARS-CoV-2)

SARS-CoV-2 is a positive-sense single-stranded RNA virus in the *Coronaviridae* family and the causal agent of coronavirus disease 2019 (COVID-19) [123]. As of September 2020, over 30 million people have been infected by SARS-CoV-2 with 1 million fatalities. SARS-CoV-2 primarily targets the respiratory tract [124] and can result in pneumonia and severe acute respiratory distress syndrome especially in the elderly and in individuals with comorbidities [124,125]. Epithelial cell death and lung inflammation are major hallmarks of SARS-CoV-2 induced tissue damage [126]. Although the clinical presentation is heterogeneous, with many mild cases, a defining feature of severe COVID-19 is a marked hypoxaemia. Recent studies show multi-organ involvement in severe COVID-19 disease [127], including the gastrointestinal tract [128] and central nervous system [127,128,129]. SARS-CoV-2 encoded Spike protein binds human angiotensin-converting enzyme (ACE2) and the transmembrane proteases [130,131], serine 2 (TMPRSS2) and furin, trigger the fusion of viral and cell membranes [130,131]. Hypoxia has been reported to reduce ACE2 expression in lung pulmonary arterial smooth muscle cells [132] and haematopoietic stem cell precursors [133] via regulating ACE1. Wing et al. showed that hypoxia and the HIF PHD inhibitor Roxadustat reduced ACE2 expression and inhibited SARS-CoV-2 entry in lung epithelial cells via a HIF-1α dependent signalling pathway [134]. Importantly, this study showed that hypoxia and pharmacological activation of HIFs inhibited SARS-CoV-2 RNA replication, showing that post-entry steps in the viral life cycle are oxygen-sensitive. This is consistent with a recent report showing a gradient of ACE2 expression in proximal (high) versus distal (low) pulmonary epithelial cells that associates with SARS-CoV-2 infection [124]. In contrast, neonatal hyperoxia increases ACE2 and TMPRSS2 expression in an age-dependent fashion [135] via a loss of type II alveolar epithelial cells. Given reports that neonatal hyperoxia associates with risk of more severe IAV infection in adults via a loss of type II alveolar epithelial cells [113,114,115], this provides an explanation for the increased severity of COVID-19 in elderly and people with pre-existing co-morbidities.

HIF-1α protein was detected in monocytes isolated from bronchoalveolar lavage and circulating neutrophils in COVID-19 patients [136,137]. These observations were validated in vitro and showed that SARS-CoV-2 infection of monocytes induced mitochondrial ROS and stabilised HIF-1α expression and associated gene transcription [136]. SARS-CoV-2 infection of human brain organoids also showed evidence of virus-dependent HIF expression [138]. In contrast, Appelberg et al. reported that SARS-CoV-2 infection of human hepatoma Huh-7 cells repressed HIF-1α expression [139], suggesting cell-type specific effects of viral infection on this pathway. Codo et al. demonstrated that pharmacological inhibition of mitochondrial ROS and associated HIF-1α activity in monocytes reduced SARS-CoV-2 infection and interleukin-1β expression in monocytes [136]. Furthermore, inhibition of HIF-1α restored T cell proliferation and rescued apoptosis in co-cultured A549 cells. These data contrast to those reported by Wing et al. who showed that treatment of lung epithelial cells with the HIF PHD inhibitor Roxadustat significantly reduced SARS-CoV-2 entry and replication [134]. These contrasting observations may reflect cell-type specific differences; for example, monocytes have limited permissivity to support SARS-CoV-2 replication and viral RNA levels were substantially lower than reported in lung epithelial cells. Collectively, these studies highlight the importance of hypoxia and HIF signalling in multiple aspects of SARS-CoV-2 life cycle, suggesting that targeting the HIF oxygen sensing pathway could offer a novel therapeutic modality for COVID-19. The diverse role of HIFs and other hallmarks of a low oxygen environment in the inflamed lung are likely to be complex and are worthy of further investigation.

## 6. Therapeutic Implications

### 6.1. HIF Modifiers

Given the important role that the PHD-HIF pathway plays in regulating the replication and associated pathogenesis of many viruses, inhibitors targeting PHD and HIF undergoing clinical trials could be repurposed for the treatment of viral diseases. Four PHD inhibitors that stabilise HIFα have been evaluated in phase II and III clinical trial studies for the treatment of anemia: Roxadustat (FG4592, FibroGen), Daprodustat (GSK1278863, GlaxoSmithKline), Molidustat (Bay8503934, Bayer), Vadadustat (AKB-6548, Akebia) [20]. Roxadustat was recently licensed for the treatment of chronic kidney disease and anemia in China [140]. Future studies could examine the therapeutic potential of these inhibitors in regulating viruses such as HIV and IAV that are repressed by HIFs.

A number of clinical trials have assessed the efficacy of HIF inhibitors in patients with advanced or refractory cancers [21]. The HIF-1α synthesis inhibitor Digoxin is undergoing phase II clinical trial for Kaposi’s Sarcoma [141]. CRLX101, a HIF-1α expression inhibitor, has completed a phase II trial in combination with bevacizumab, an antibody that targets VEGF, for the treatment of recurrent platinum-resistance ovarian, tubal, and primary peritoneal cancers and reduce tumor frequency and size [142,143]. However, a randomised phase II trial in patients with advanced renal carcinoma did not show any improvement in progression-free survival [144]. A phase II trial for CRLX101 in combination with capecitabine and radiotherapy for locally advanced rectal cancer is ongoing [145]. The HIF-2α specific dimerisation inhibitors PT2385 [146,147] and PT2977 [148,149] are being tested for the treatment of recurrent glioblastoma, non-metastatic VHL-associated and advanced clear cell renal carcinoma [149,150,151]. Viruses known to be positively regulated by HIF-2α, including KSHV and HCV could benefit from clinical trials focusing on HIF inhibitors. Furthermore, viruses whose pathogenesis has been linked to HIF-signalling, including HBV, HCV, HPV, EBV, KSHV and SARS-CoV-2 may benefit from HIF inhibitors. Since KDM5 demethylases induce a robust interferon response resulting in an increased resistant to infection, the first KDM5 inhibitor entered a phase I clinical trial for the treatment of HBV infection [152,153].

### 6.2. Engineered Oncolytic Viruses

In addition to direct targeting the PHD-HIF pathway, a number of studies have engineered oncolytic viruses to encode HRE to promote their replication in hypoxic tumour environments as anti-cancer agents. Post et al. inserted a HRE within the E1A promoter of the adenovirus genome and demonstrated enhanced viral-mediated cytolysis of human brain tumour cells under hypoxic conditions [154]. A similar study using an adenovirus expressing E1A under the control of a HRE showed preferential lysis of hepatoma, pancreatic cancer and lung tumour cell lines under hypoxic conditions [155]. The authors reported that the recombinant viruses resulted in a significant survival improvement in a nude mice xenograft model of prostate cancer. As hypoxia commonly occurs in solid tumours, such targeted genetic engineering of oncolytic viruses represents a promising novel cancer treatment modality.

Viruses that are used for vaccination purposes may also regulate HIF pathways. Vaccinia virus (VACV) has traditionally been used as a vaccine against smallpox and stabilise HIF expression via the C16 protein [156]. The N-terminal region of C16 binds and inhibits the human oxygen sensor, PHD2, resulting in HIF expression under normoxic conditions. Mazzon et al. compared the metabolic alterations of cells infected with wild type VACV or a mutant lacking C16 and found a role for C16 in regulating nucleotide, glucose and glutamine metabolic pathways [157]. C16 has homologs in other poxviruses, suggesting an evolutionary conserved role in their replication pathways [156]. Another example of a virus that modulates HIF signaling is the mammalian orthoreovirus [158,159,160,161]. This oncolytic virus has completed phase I-III clinical trials against many different types of cancer [162]; yet little is known about the specific mechanisms involved and further studies will greatly enrich therapeutic options.

## 7. Concluding Statement

This review has illustrated how variable oxygen tension in different tissues can affect many stages of the virus life cycle: regulating entry receptors, replication machinery, particle genesis and host-pathogen interactions (Table 1). Current information suggests that hypoxia preferentially enhances the replication of viruses with a tropism for low oxygen environments, whereas HIFs can dampen the replication of viruses that replicate in tissues with higher oxygen levels. Differences in oxygen tension and associated HIF signaling may play an important role in viral tropism and pathogenesis. Viruses have evolved to replicate in a tissue specific oxygen environment and developed ways to manipulate the metabolic micro-environment to their advantage. Given their wide-ranging effects on cellular metabolism low oxygen environments may influence the efficacy of both antiviral agents and immune based therapies and is worthy of further study. In vitro cell-based systems that utilise physiological oxygen tension may provide improved pre-clinical models for evaluating new anti-viral agents. Further studies to understand these mechanisms are crucial for generating novel or repurposing existing treatment strategies for viral infection and pathogenesis.

## Figures and Tables

**Table 1:** Mechanisms in which viruses interact with HIFs.

Virus	Viral Component	Mechanism Categorisation	Mechanism Description	Citation
KSHV	G protein-coupled receptor (vGPCR)	Direct interaction	- Triggers HIF-1a phosphorylation via p38 and MAPK pathways - Upregulates HIF-1α and HIF-2α proteins via mTOR pathway modulation	[30]
	Latency-associated nuclear antigen (LANA)	Direct interaction	- Directly interacts with HIF-1α to enhance binding to HIF-1α promoter and upregulate HIF-1α mRNA	[32,33]
	Viral IFN regulatory factor 3 (vIFR3)	Direct interaction	- Stimulates HIF-1a transcription and protein stability by blocking HIF-1α degradation	[31]
HPV	HPV16 E6 and E7 proteins	Direct interaction	- Regulate HIF-2α protein via LKB1 modulation	[43]
	HPV16 E6	Direct interaction	- Prevents HIF-1α and VHL binding and reduces HIF-1α ubiquitination to upregulate HIF-1α protein	[42]
	HPV18 E2	Indirect interaction	- Upregulates ROS by binding to inner mitochondrial membrane components of the respiratory chain to stabilise HIF-1α	[44]
HCV	HCV virus	Indirect interaction	- Oxidative stress stabilises HIF-1α protein via NF-kB, STAT-3, PI3-K-aKT, and p42/44 MAPK pathway dependent mechanisms	[54,59,60,61]
	Core protein	Indirect interaction	- Regulates HIF-1α protein but not HIF-2α by unfolded protein-stress response	[55,56,57,58]
	E1E2 Glycoproteins	Indirect interaction	- Regulates HIF-1α protein by unfolded protein-stress response	[52]
HBV	HBx protein	Direct interaction	- HIF-1α protein and mRNA upregulation through: (1) upregulation of MTA1 and HDAC1/2 which perturbed HIF-1α deacetylation and (2) prevention of HIF-1α association with VHL and down-stream degradation	[66,67,68]
	HBV virus	Indirect interaction	- Inflammation and oxidative stress induced by viral infection can upregulate transcriptional activity of HIF	[71]
HIV	HIV virus, Viral protein R (Vpr)	Indirect interaction	- ROS induction increases HIF-1α mRNA and protein	[83,84,85,163]
HTLV-1	Transactivator protein (Tax)	Direct Interaction	- induces HIF-1α protein expression	[88]
EBV	Latent membrane protein 1 (LMP1)	Indirect interaction	- HIF-1α protein degradation is inhibited via proteasomal degradation of PHD1 and PHD3 through Siah1 E3 ubiquitin ligase upregulation - additionally, the loss of VHL/HIF-1α complexes stabilises HIF-1α and associated HIF signaling	[89,92,93,94,95]
DENV	DENV	Indirect interaction	- induces an oxidative stress response that might lead to HIF stabilisation and hypoxic reprogramming	[104]
IAV (PR8)	IAV	Direct interaction	- FIH-1 inhibition leads to a reduction in HIF-1a degradation	[119]
		Indirect interaction	- Inflammatory responses (including acute lung injury) activate hypoxia signalling and HIF induction - Increased HIF-1α nuclear translocation	[121]
SARS-Cov-2	SARS-Cov-2 virus	Indirect interaction	- Induces mitochondrial ROS and upregulates HIF-1α protein	[136,137,138]
